# The Role of Ctk1 Kinase in Termination of Small Non-Coding RNAs

**DOI:** 10.1371/journal.pone.0080495

**Published:** 2013-12-04

**Authors:** Tineke L. Lenstra, Agnieszka Tudek, Sandra Clauder, Zhenyu Xu, Spyridon T. Pachis, Dik van Leenen, Patrick Kemmeren, Lars M. Steinmetz, Domenico Libri, Frank C. P. Holstege

**Affiliations:** 1 Molecular Cancer Research, University Medical Center Utrecht, Utrecht, The Netherlands; 2 LEA Laboratory of Nuclear RNA Metabolism, Centre de de Génétique Moléculaire, C.N.R.S.-UPR3404, Gif sur Yvette, France; 3 Genome Biology Unit, European Molecular Biology Laboratory, Heidelberg, Germany; University of Edinburgh, United Kingdom

## Abstract

Transcription termination in *Saccharomyces cerevisiae* can be performed by at least two distinct pathways and is influenced by the phosphorylation status of the carboxy-terminal domain (CTD) of RNA polymerase II (Pol II). Late termination of mRNAs is performed by the CPF/CF complex, the recruitment of which is dependent on CTD-Ser2 phosphorylation (Ser2P). Early termination of shorter cryptic unstable transcripts (CUTs) and small nucleolar/nuclear RNAs (sno/snRNAs) is performed by the Nrd1-Nab3-Sen1 (NNS) complex that binds phosphorylated CTD-Ser5 (Ser5P) *via* the CTD-interacting domain (CID) of Nrd1p. In this study, mutants of the different termination pathways were compared by genome-wide expression analysis. Surprisingly, the expression changes observed upon loss of the CTD-Ser2 kinase Ctk1p are more similar to those derived from alterations in the Ser5P-dependent NNS pathway, than from loss of CTD-Ser2P binding factors. Tiling array analysis of *ctk1*Δ cells reveals readthrough at snoRNAs, at many cryptic unstable transcripts (CUTs) and stable uncharacterized transcripts (SUTs), but only at some mRNAs. Despite the suggested predominant role in termination of mRNAs, we observed that a *CTK1* deletion or a Pol II CTD mutant lacking all Ser2 positions does not result in a global mRNA termination defect. Rather, termination defects in these strains are widely observed at NNS-dependent genes. These results indicate that Ctk1p and Ser2 CTD phosphorylation have a wide impact in termination of small non-coding RNAs but only affect a subset of mRNA coding genes.

## Introduction

Transcription termination is essential for generating normal transcripts and for the release of RNA Polymerase II (Pol II) from the DNA template, which prevents interference between adjacent transcription units. Transcription termination also plays an important role in the control of pervasive and hidden transcription. In yeast, transcription of mRNA coding genes is terminated by the Cleavage and Polyadenylation Factor/Cleavage factor I and II (CPF/CFI-II) that also cleaves the nascent RNA and polyadenylates the released transcript. The Nrd1p-Nab3p-Sen1p (NNS) complex terminates transcription of sn-/snoRNAs and cryptic unstable transcripts (CUTs), one of the main products of hidden transcription. NNS-dependent termination is tightly coupled to nuclear RNA processing by the Rrp6p/exosome complex [1], [2], which leads to trimming of primary sn-/snoRNA transcripts and full degradation of CUTs. Thus, the choice of termination pathway is essential to determine the fate of transcripts, which are stable and can be exported for translation in the case of the CPF/CFI-II pathway but are generally unstable when generated by the NNS pathway [1]–[4].

The carboxy-terminal domain (CTD) of Rpb1 subunit of Pol II [5], [6] is thought to play an important role in the coordination of co-transcriptional events including transcription termination. The CTD is conserved across eukaryotes, is essential for viability, and consists of multiple repeats of the hepta-peptide sequence Tyr1-Ser2-Pro3-Thr4-Ser5-Pro6-Ser7. The number of repeats varies between organisms, and is 26 in yeast. The CTD is modified during transcription, thereby forming a dynamically changing binding platform for appropriate recruitment of regulatory factors [7]. Upon Pol II recruitment to promoters, the CTD is hypophosphorylated, but early after transcription initiation, the repeat is phosphorylated on Ser5 and Ser7. While Pol II progresses into the gene body, Ser5 phosphorylation (Ser5P) is removed. The opposite pattern is found for Ser2P, which is low at the transcription start site and gradually increases as Pol II reaches the end of the transcript [8]–[11]. The major Ser2P kinase is Ctk1p, but Bur1p has also been shown to contribute to Ser2P near the promoter [12], [13]. Ser5P is added by Kin28p, a component of the TFIIH general transcription factor.

The Ser2/Ser5 phosphorylation gradients have been proposed to form a ‘CTD code’, sequentially recruiting proteins important for efficient transcription and co-transcriptional chromatin modification [7], [14]. For example, CTD-Ser5P interacts with the capping enzyme, the histone methyltransferase Set1p, while CTD-Ser2P associates with the histone methyltransferase Set2p [15]–[21].

The CTD phosphorylation status is also thought to direct the choice between the two main yeast transcription termination pathways [21]–[23]. During transcription, the position of Pol II relative to the transcription start site specifies a defined phosphorylation pattern (Ser5P to Ser2P ratio), which is thought to regulate the choice of the termination pathway. The NNS pathway has been shown to better function within a window of 1 kB from the transcription start site [23], [24], which is mechanistically underlain by the preferential interaction of the NNS component Nrd1p with CTD-Ser5P [21], [25]. Longer transcripts and most of the mRNAs are usually terminated by the action of the CPF/CF complex and the Rat1p complex, which contain the Ser2P-binding subunits Pcf11p and Rtt103p [16], [20], [22]. Both termination pathways also rely on the presence of termination signals on the nascent RNA that are recognized by distinct RNA binding components of each complex (e.g. Nrd1p and Nab3p in the case of the NNS pathway).

The distinction between the early and late termination pathways may not be strict, since several factors, including Pcf11p, appear to be involved in termination by both the “early” and “late” pathway [22]. Indeed, it has been proposed that transcription units have a preference for either pathway (determined by RNA sequence motifs and CTD status), but at a given site the pathways are not exclusive [7]. It is unclear, however, whether shared factors partake in both pathways or whether the CPF and the NNS complexes independently recognize both CUTs/snoRNA and mRNA terminators.

By contributing to the establishment of the CTD phosphorylation gradient, the CTD-Ser2 kinase Ctk1p is thought to influence the choice between the termination pathways. Loss of Ser2P affects the recruitment of late termination factors, such as Pcf11p and Rtt103p. Surprisingly however, a 3′-processing but not a termination defect has been observed at a CPF-dependent model gene in *ctk1Δ* cells [26]. Ctk1p has also been reported to be required for correct co-transcriptional snoRNP assembly at several H/ACA snoRNAs, which in turn was shown to be required for both accumulation of mature snoRNA and efficient termination [27], [28]. However, the precise function of Ctk1p in the early termination pathway and whether it relates to CTD-Ser2 phosphorylation remains unclear.

To investigate the role of Ctk1p systematically, we performed genome-wide expression analysis of Ctk1p mutants and several CTD binding factors. Surprisingly, the overall expression changes of *ctk1*Δ were much more similar to mutation of the NNS-complex subunit Sen1p, than to mutation of CTD-Ser2P binding factors. Genome-wide tiling array analysis of *CTK1* deletion shows readthrough at 42% of all snoRNAs, as well as readthrough at several CUTs and stable uncharacterized transcripts (SUTs). Unexpectedly we also found that a *ctk1*Δ strain does not present a major defect in mRNA termination as we only detected transcriptional readthrough at a few mRNA-coding genes. Our results show that the role of Ctk1p in early termination is more prominent than previously thought and suggest a significant impact of Ser2 phosphorylation on the mechanism of NNS-dependent termination.

## Materials and Methods

### Yeast strains and plasmids

Yeast strains used in this study are listed in Table S2. Plasmids were described previously [29], [30]. Construction of strains containing *CTK1* or *BUR1* under the control of the *GAL1* promoter was performed as described [31].

### Northern blot analysis

RNA was isolated by phenol extraction from cultures grown to early mid-log phase. Northern blot analysis was performed using standard protocols. Hybridization was performed either in 6× SSC, 5× Denhardt's solution, 0.1% SDS and 100 µg/nl fragmented haring sperm DNA buffer or in UltraHyb buffer (Ambion). Quantification of the signals was carried out using a Storm 820 phosphoimager. Oligonucleotide probes and primers used to amplify the PCR probes are listed in Table S3.

### Protein analyses and immunoprecipitations

For immunoprecipitations, cells were grown to OD 1.0–1.6 and harvested. The cell pellet was resuspended in 4 ml of Lysis buffer (6 mM Na_2_HPO_4_, 4 mM NaH_2_PO_4_, 200 mM sodium acetate, 0.25% NP-40, 2 mM EDTA, 1 mM EGTA, 5% glycerol, 20 mM PMSF, 1 mM benzamidine, EDTA-free protease inhibitor cocktail from Roche) and flash frozen. Cells were disrupted using a ball mill (MM400 Retsch; 5 times 10 Hz, 3 min) and the extract cleared at 13krpm for 25 min. Immunoprecipitation assays were performed using IgG Fast Flow Sepharose beads (GE Healthcare). Proteins were eluted for 15 min at 37°C with 60 µl of lysis buffer and 80 µl 4× Laemmli buffer containing 50 mM DTT. RNAseA treatment was performed after IP on beads washed once with 1 ml Lysis buffer (10 µg/ml RNaseA for 20 min in 25°C).

To analyze the phosphorylation status of RNA Pol II CTD after Ctk1p and Bur1p depletion, 4OD units of exponentially growing cells were harvested after growth in the appropriate medium and resuspended in 150 µl of 8 M urea. Cells were broken in a Magnalyser (Roche) and the lysate was cleared by centrifugation for 2 min at 4000 rpm. Alternatively, exponentially growing *ctk1*Δ and wt cells were harvested and disrupted in FA lysis buffer (50 mM Hepes KOH pH 7.5, 150 mM NaCl, 1 mM EDTA, 1% Triton X-100, 0.1% Deoxycholate, 0.1% SDS), containing protease and phosphatase inhibitors using a genie-disruptor (Scientific Industries). Western blot analysis was performed using antibodies 8WG16 (gift from H.T.M. Timmers), 3E8, 3E10, 4E12 (Millipore) [32], Tub1p (Immunologicals), H5, H14 (Covance), Y-80 (Santa Cruz), 12ca5 (Roche), αSpt5 (gift from G. Hartzog), αNab3p (gift from M. S. Swanson), αRbp3p (Neoclone), α-Nrd1p (gift from D. Brow).

### Expression profiling

Each mutant strain was profiled four times from two independently inoculated cultures and harvested in early mid-log phase in SC medium with 2% glucose. An exception is Pcf11, for which two different alleles (*pcf11-2* and *pcf11-ts2*) were profiled two times from single inoculated cultures. Sets of mutants were grown alongside corresponding wt cultures and processed in parallel. Growth of *ctk1*Δ and *rpb1-S2A* for Figure 5 was performed in YPD, because the *rpb1*-*S2A* strain grew very poorly in SC. Dual-channel 70-mer oligonucleotide arrays were employed with a common reference wt RNA. All steps after RNA isolation were automated using robotic liquid handlers. These procedures were first optimized for accuracy (correct fold-change) and precision (reproducible result), using spiked-in RNA for calibration [33]. After quality control, normalization, and dye-bias correction [34], statistical analysis for mid-log cultures was performed for each mutant versus the collection of 200 wt cultures as described previously [35]. The temperature sensitive mutants of *SEN1* and *PCF11* were compared to wt Mat a strains. The reported fold-change is an average of the replicate mutant profiles versus the average of all wildtypes. Microarray data have been submitted to the ArrayExpress database (http://www.ebi.ac.uk/microarray) under accession number E-MTAB-1261 and to the GEO database (http://www.ncbi.nlm.nih.gov/geo/) under accession number GSE40254

### Genome-wide analyses

Tiling arrays were performed for two independent replicates of wt and *ctk1Δ* as described previously [36], [37]. The occurrence of readthrough at ORFs and snoRNAs was assessed as follows: the probe intensities from the two replicates were averaged over 25 bp regions and aligned to the 3′ end of ORFs and snoRNAs. Readthrough was scored positive if the expression difference between *ctk1Δ* and wt was more than 0.6 in the 150 bp regions downstream of the 3′ end (at least 4 out of 6 25 bp regions). Transcripts for which the 3′ end was less than 150 bp from the following transcript were excluded. Metagene analysis was performed by averaging the intensity over 25 bp regions for all ORFs and snoRNAs. For visualization in Figure 2, the tiling data was segmented separately for wt and *ctk1*Δ using the library tilingArray in R [36], [37]. The optimal number of segments was selected based on the maximum log likelihood (BIC). The data can be visualized for individual genes at http://steinmetzlab.embl.de/holstegeLabArray/.

### Chromatin Immunoprecipitation

500 ml of cultures in early mid-log phase were crosslinked with 1% formaldehyde for 20 min at 25°C. The reaction was stopped with 300 mM glycine. Cells were washed with ice-cold TBS and FA lysis buffer (50 mM Hepes KOH pH 7.5, 150 mM NaCl, 1 mM EDTA, 1% Triton X-100, 0.1% Deoxycholate, 0.1% SDS). Cells were disrupted in FA-lysis containing protease and phosphatase inhibitors using a gene-disruptor. Chromatin was fragmented by sonication (Bioruptor, Diagenode: 7 cycles, 30 sec on/off, medium setting), resulting in fragment sizes of 300–500 bp. Chromatin extracts were incubated overnight at 4°C with antibody (1 µl α-Nrd1, 1 µl α-rpb3 (Neoclone), 2 µl 3E8, 2 µl 3E10, 10 µl 4E12). 20 µl of dry protein A (for α-Nrd1) or protein A+G beads (for all other antibodies) were added and incubated for 1.5 hr at RT. For TAP-Pcf11, 20 µl of IgG sepharose beads were used. The beads were washed 2× with FA-lysis buffer, 2× with FA lysis buffer containing 500 mM NaCl, 2× with 10 mM Tris pH 8, 0.25 M LiCl, 0.5% Nonidet P-40, 0.5% sodium deoxycholate, 1 mM EDTA and 1× with TE pH 8.0. Samples were eluted with TE-SDS 1% at 65°C. Crosslinking was reversed at 65°C for ∼16 hr with 5 µg RNAse. Samples were treated with Proteinase K for 2 hr at 37°C. DNA was recovered with phenol extraction and a PCR purification kit (Qiagen). Samples were analyzed by qPCR with the primer pairs in Table S3. Every experiment is based on at least 2, in most cases 4 biological replicates and the signals represent enrichment over input. Mock ChIPs contain no antibody. For mock ChIP of Pcf11, untagged strains were used.

## Results

### The expression signature of *ctk1*Δ is most similar to loss of an NNS complex component

**Figure 1:**
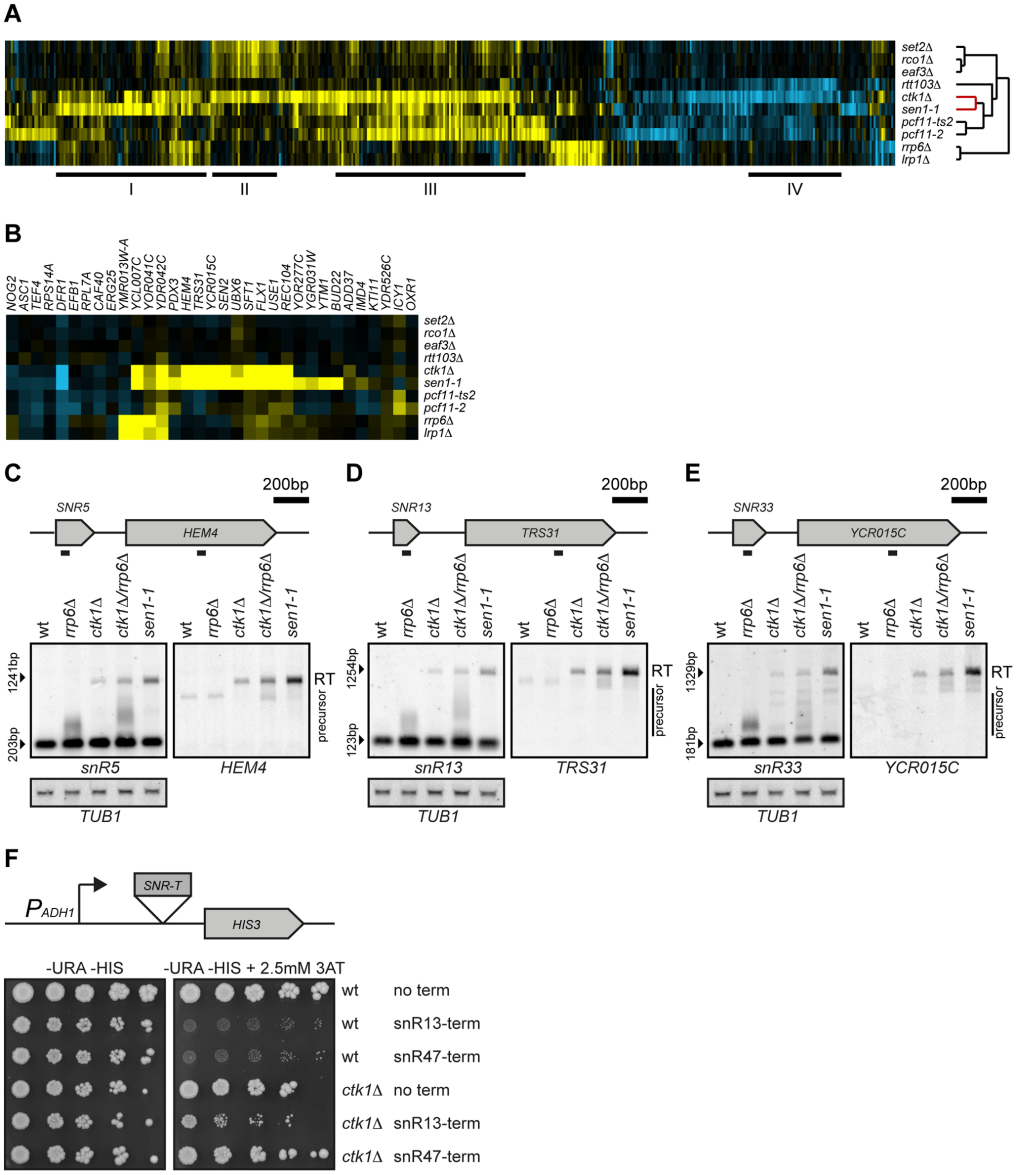
Loss of *CTK1* results in readthrough at snoRNAs. (A) Unsupervised hierarchical cluster diagram (cosine correlation) of expression changes in mutants of Ctk1p and several CTD-binding factors. All genes with significant changes (*p*<0.05, FC>1.7) in any of the mutants individually are shown, yellow indicating upregulation, blue indicating downregulation, and black indicating no change compared to wt. The dendrogram indicates the similarity between the mutants. The relationship between Ctk1p and Sen1p is highlighted in red. Overlapping expression changes between *ctk1*Δ and other mutants are underlined and marked by I (*ctk1*Δ, *sen1-1*), II (*ctk1*Δ, *set2*Δ, *rco1*Δ, *eaf3*Δ), III (*ctk1*Δ, *pcf11-2*, *pcf11-ts2*), IV (*ctk1*Δ, *rtt103*Δ). (B) Expression changes for the first gene that is positioned within 2 kB of a snoRNA termination site. Upregulation suggests defective termination at the snoRNA. (C, D and E) Northern blot analysis at *snR5*, *snR13*, and *snR33*. The positions of the probes are indicated on the scheme above the Northern blots. *TUB1* mRNA is used as a loading control. Precursors and readthrough transcripts (RT) are indicated. (F) Wt and *ctk1*Δ cells were assessed for their ability to express the *HIS3* genes, which was positioned on a plasmid containing no terminator, or the terminators of *snR13* or *snR47* upstream of the *HIS3* gene. Expression of *HIS3* is reflected by the ability to grow on plates containing the histidine analogue 3-AT, and indicates readthrough. Serial dilutions (1∶5) are shown starting at 1*10^4^ cells after growth for 5 days on SC -URA -HIS and SC -URA -HIS+ 2.5 mM 3-AT plates.

Expression profiles that describe the genome-wide changes in gene expression upon mutation of different regulatory factors can provide detailed phenotypical information. For example similarities between the genome-wide expression signatures derived from mutation of different factors can indicate different types of interactions, including same protein complex or regulatory pathway membership [35], [38]. To assess the role of the CTD-Ser2 kinase Ctk1p in early and late termination pathways, genome-wide expression changes of a *ctk1Δ* mutant were compared with expression changes of mutants of different CTD-interacting factors (Figure 1A). For each factor, we determined the expression profile in quadruplicate (two technical replicates from two independent cultures), averaged and statistically analyzed versus many wild type strains grown and processed identically (Materials and Methods). Most temperature-sensitive (ts) strains already show partial loss of function at the permissive temperature [39]. To ensure data comparability, all strains, including the ts strains for the essential genes *SEN1* and *PCF11*, were grown under identical conditions including temperature (30°C).

Figure 1A shows all genes with a significant change in expression in any mutant as compared to wild type (*p*<0.05, fold change (FC)>1.7). In keeping with previous observations that similar expression profiles may reflect interactions, loss of Ctk1p results in mRNA expression changes that overlap with changes observed upon mutation of several different binding partners of CTD-Ser2P. For example, the expression changes in *ctk1*Δ overlap with changes observed upon deletion of the Ser2P binder Set2p (Figure 1A, II, Table S1). Upon binding to Ser2P, Set2 trimethylates histone 3 on lysine 36 in the coding region of actively transcribed genes [17], [19], which leads to activation of Rpd3S complex and deacetylation of coding regions, preventing initiation of cryptic transcription in gene bodies [40]–[43]. Removal of Ctk1p, Set2p and the downstream components of Rpd3S complex, Eaf3p and Rco1p, all result in elevated expression levels of a similar set of genes (Figure 1A, II). As expected, this set includes genes with internal transcription initiation sites, such as *STE11* [40] (Table S1). Besides reflecting this previously known interaction between Ctk1p and Set2p, loss of Ctk1p also results in changes that overlap with mutants of the Ser2P binding factors Rtt103p and Pcf11p (Figure 1A, III and IV, Table S1). Gene set III is significantly enriched for binding sites of the gene specific transcription factors Msn2p, Msn4p and Gcn4p, which regulate stress and amino acid biosynthesis genes. Gene set IV, shared primarily between *ctk1*Δ and *rtt103*Δ, is enriched for genes with weak polyA sites (hypergeometric test, p<2.82*10^−4^) [11], suggesting that their decrease might be caused by instability after readthrough. Both mutants result in defects in the late termination pathway that acts on mRNA genes [20], [22].

The dendrogram on the right side of figure 1A reflects overall similarities between expression signatures and is automatically generated using hierarchical clustering with no input of prior knowledge. Surprisingly, the overall expression profile of *ctk1*Δ is more similar to *sen1-1*, a member of the Ser5P binding NNS-complex, than to any of the Ser2P binding factors (Figure 1A, red part of tree). The expression changes of *ctk1*Δ and *sen1-1* cells show a large overlap (Figure 1A, I, Table S1), of which some transcripts are also affected in mutants of the nuclear exosome, *rrp6*Δ and *lrp1*Δ, involved in degradation and trimming of NNS-dependent transcripts. The large overlap between *sen1-1* and *ctk1Δ* suggests a prominent role of Ctk1p in early termination.

### Defective termination at snoRNAs in ctk1Δ cells

One class of transcripts terminated by the early termination pathway consists of snoRNAs [22], [27]. Detailed analysis of the genes with altered expression in both *ctk1*Δ and *sen1-1* reveals that many are positioned downstream of snoRNAs, suggesting that transcriptional readthrough from the upstream snoRNA might artificially increase the signal downstream. Previous studies have indeed reported readthrough at a few model snoRNAs in *ctk1Δ* [27], [28], although whether this reflects a direct or indirect effect mediated by Ser2 phosphorylation of the CTD was unclear.

Deletion of *CTK1* appears to lead to the upregulation of the first gene following a snoRNA terminator in approximately one third of cases. All of these genes are also upregulated in *sen1-1* (Figure 1B). To confirm that these transcripts originate from defective snoRNA termination, RNA from *ctk1*Δ cells was analyzed by Northern blot. A double mutant of *ctk1*Δ and *rrp6*Δ was included to avoid transcripts being missed due to nuclear degradation. In *ctk1*Δ, *ctk1*Δ/*rrp6*Δ, and *sen1-1*, a long *snR5* transcript is observed that also hybridizes to a probe for the downstream gene *HEM4* (Figure 1C), indicating the presence of a fusion transcript between *snR5* and *HEM4*. Similar fusion transcripts are also observed for *snR13*, *snR33*, *snR43*, *snR60* and *snR82* (Figure 1D, 1E, Figure S1). The same fusion transcripts are observed in *sen1-1*, showing that these transcripts are caused by defective termination at the snoRNA termination sites. Although the termination defect is not as strong as for the *sen1-1* mutant (note that this analysis is performed at permissive temperature and that only a partial termination effect occurs in *sen1-1* cells), it is clear that integrity of Ctk1p is nevertheless important for appropriate termination of snoRNAs.

In all these cases we also noticed that the snoRNA precursor (only visible in double *ctk1Δ/rrp6Δ* mutants when its conversion to the mature form is less efficient) migrates anomalously, with longer forms that extend in some cases for several hundred bases. These transcripts likely derive from delayed termination events that in most cases depend on the NNS pathway, since they are degraded in the wild type strain.

Ctk1p has been shown to influence the co-transcriptional assembly of snoRNAs, which, in turn, might affect termination [28]. It is also possible that the lack of Ser2P alters the local chromatin context, which could impact termination. Therefore we assessed the efficiency of termination using a reporter system in which a snoRNA terminator sequence is inserted upstream of the *HIS3* gene and prevents its expression unless transcriptional readthrough occurs. This construct misses sequences directing the co-transcriptional assembly of the core particle and has previously been used to analyze *cis* and *trans* elements that direct termination of snoRNAs [29], [30]. As expected, wild type cells are inhibited in growth in the absence of histidine if either *snR13* or *snR47* termination sequences are placed upstream of the *HIS3* marker. In contrast, *CTK1* deletion mutants show near-normal growth with the *snR47* reporter and only partial inhibition of growth with the *snR13* reporter, indicating readthrough at both sites (Figure 1F). These results indicate that the readthrough phenotype observed in *ctk1Δ* cells is linked to inefficient recognition and/or use of snoRNA termination signals, independent of the genomic context. The generality and the mechanism of Ctk1p dependency were therefore further investigated.

### Widespread termination defects at NNS-dependent genes and minor defects at mRNA coding genes in *ctk1Δ* mutants

**Figure 2:**
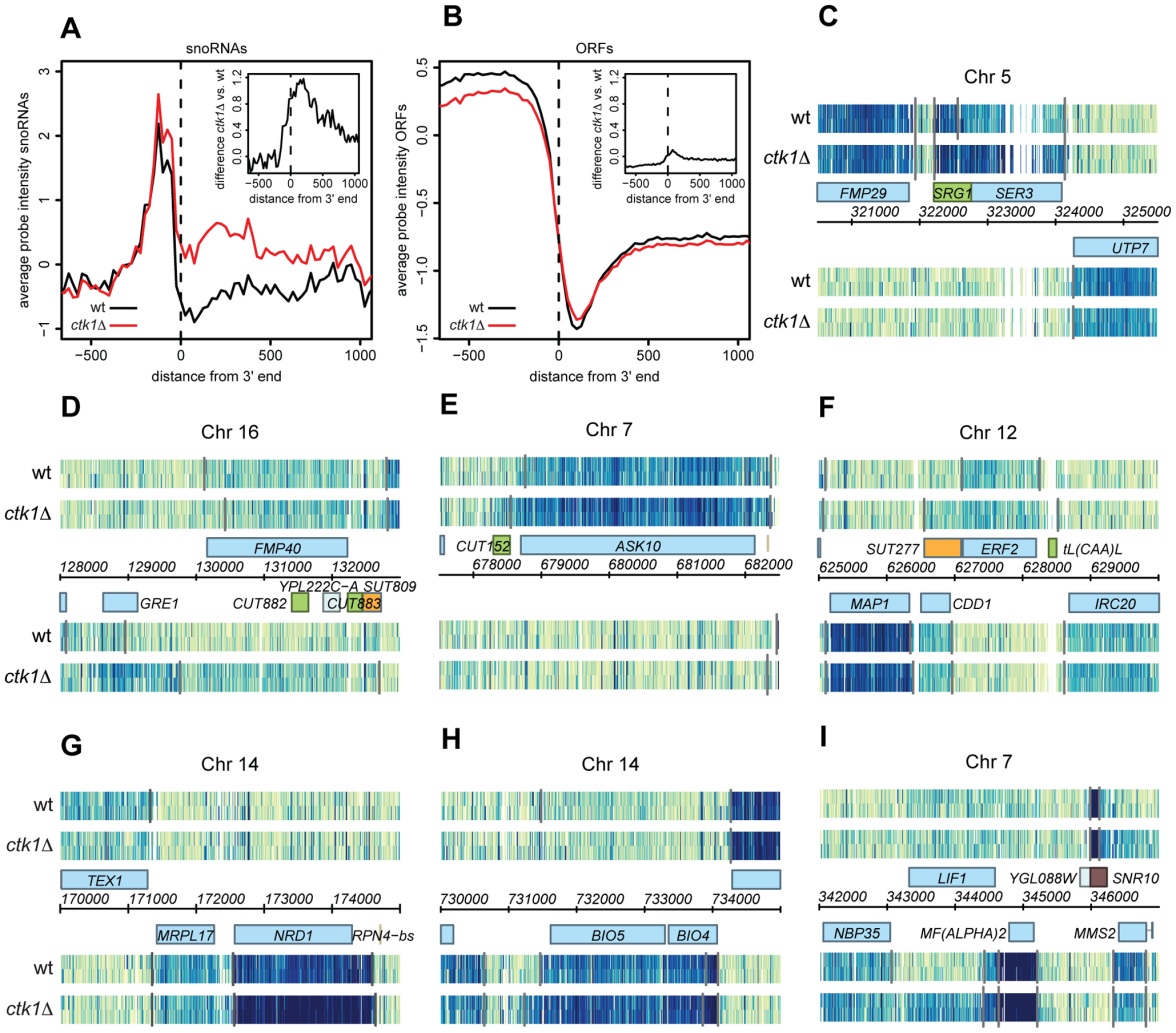
Loss of *CTK1* also results in termination defects at several upstream regulatory ORFs, CUTs and coding genes. Expression analysis using tiling arrays in two replicate cultures of wt and *ctk1*Δ. (A–B) Metagene analyses displaying the average probe intensity over 25 bp regions in wt and *ctk1*Δ, aligned by the 3′ end of the mature snoRNAs (A) or ORFs (B). Insets show the difference in expression between *ctk1*Δ and wt. (C–I) Examples of readthrough at non-coding and coding genes: (C) *SRG1*-*SER3*, (D) *CUT882*-*GRE1*, (E) *CUT152*-*ASK10*, (F) *SUT277-ERF2* (G) *NRD1* (H) *BIO4*-*BIO5*, (I) *MF(ALPHA)2*. Note also the readthrough at *snR10* in (I). Expression data are displayed along the chromosome (*x* axis) for the Watson (W, upper half) and the Crick (C, lower half) strands. Normalized signal intensities (higher in darker blue) are shown for 2 replicates of wt and *ctk1*Δ (*y* axis). Grey lines indicate segment boundaries as described in materials and methods. Genome annotations are shown in the center: annotated ORFs (blue boxes), SUTs (orange boxes), CUTs/non-coding RNAs (green boxes), and snoRNAs (brown). Note that CUTs and snoRNA precursors are normally not detectable in wt cells, but the occurrence of a readthrough allows detection because the longer RNAs escape degradation. Readthrough is confirmed by Northern blot for *SRG1*, *CUT882* and *BIO5* (Figure S2).

The NNS-dependent early termination pathway also terminates other non-coding transcripts including CUTs [1], [2] and several SUTs [44]. The microarrays used in the previous analysis mainly carry probes for detection of mRNAs. To assess how general the role of Ctk1p is in NNS-termination, RNA from *ctk1*Δ and wild type cells were analyzed on whole-genome tiling arrays [36], [37] (Figure 2). Although reliable detection of cryptic transcripts requires the use of degradation defective strains [3], we reasoned that some extended transcripts would be easily revealed when terminated at downstream CPF/CF sites and therefore stabilized as for snoRNA precursors [45]. Metagene analysis of hybridization signals observed around snoRNAs clearly showed increased average intensity in *ctk1Δ* cells downstream of the 3′ end (Figure 2A). In addition to snoRNAs, *ctk1*Δ also shows readthrough of several other sites (Figure 2 C–I). These readthrough sites include CUTs and SUTs, and also non-coding transcripts that lie upstream of coding genes and have been shown to have regulatory potential. For example, the upstream regulatory RNA of *SER3*, *SRG1*, shows readthrough into *SER3* in *ctk1*Δ (Figure 2C) [46]. Similar readthrough in *ctk1*Δ is observed at *CUT882* (or one of the more upstream CUTs/SUTs), *CUT152* and *SUT277*, upstream of the *GRE1*, *ASK10*, and *ERF2* genes, respectively (Figure 2D, E, F). Early termination at the *NRD1* gene, which is known to be dependent on the NNS complex [1], is also affected in *ctk1Δ* cells, as indicated by the increase in full length *NRD1* mRNA abundance (Figure 2G). Termination defects were confirmed by Northern analysis at *SER3* and *CUT882* (Figure S2) although in the latter case the readthrough appears to be unstable and only detected in the *ctk1Δ*/*rrp6Δ* double mutant (Figure S2C). Note that, as for snoRNA precursors, longer forms are observed for CUT882 in *ctk1Δ/rrp6Δ* cells, again suggesting the occurrence of delayed termination.

Ser2P phosphorylation is thought to be important for the recruitment of the CPF/CF complex, because of the interaction of Rtt103p and Pcf11p with the CTD-Ser2P. Surprisingly, we did not observe a global defect in mRNA termination upon loss of the major Ser2P kinase Ctk1p. Indeed some readthrough is observed at a set of mRNAs, but termination by the CPF/CF complex is not affected in general (Figure 2B). When the expression difference between *ctk1Δ* and wt was calculated downstream of ORFs, only 2.8% of all ORFs show clear indications of readthrough, while similar analysis for snoRNAs reveals readthrough at 32 out of 77 sites (42%). This was confirmed by northern analysis for a few model genes in a *ctk1*Δ strain (Figure S3) and by metagene analyses of the hybridization signal for ORFs and snoRNAs (Figure 2A–B and S2E–F). This result is seemingly at odds with the finding that mutation of CPF/CF components (the recruitment of which is strongly dependent on Ctk1p [47]) has a generally dramatic effect on termination of mRNA genes ([48], [49]; and data not shown). Examples of mRNA coding genes with readthrough are *BIO4/5* and *MF(ALPHA)2* (Figure 2H, I). Readthrough was not specifically observed at short (<600 nt) or long (>3 kB) mRNA coding genes (Figure S2E, F). Moreover, it cannot be excluded that the NNS-pathway contributes to termination at some mRNA coding genes as it was shown for BIO4/5 [1].

Together these experiments reveal that the absence of Ctk1p has a general impact in termination of snoRNAs and likely other NNS-dependent transcripts but only affects termination of a subset of mRNA coding genes.

### Readthrough is kinase-dependent and partially explained by Ser2 phosphorylation

**Figure 3:**
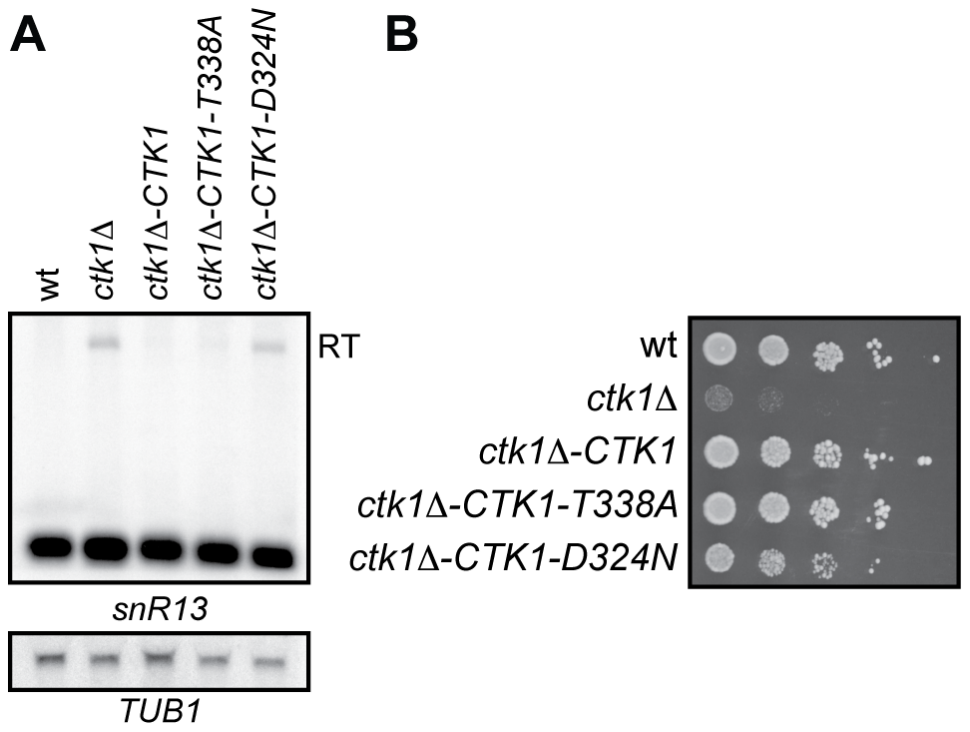
Readthrough is kinase-dependent. (A) Northern blot analysis of *ctk1*Δ with a genomically integrated copy of *CTK1-wt*, *CTK1-T338A*, or *CTK1-D324N*. Ctk1-D324N is kinase-dead (data not shown) and cannot rescue the readthrough. (B) Growth of *ctk1*Δ with a genomically integrated copy of *CTK1-wt*, *CTK1-T338A*, or *CTK1-D324N*. Ctk1-D324N is kinase-dead (data not shown) and can partially rescue the growth defect of *ctk1*Δ. Serial dilutions (1∶5) are shown starting at 1*10^4^ cells after growth for 2 days on YPD plates at 30°C.

We first assessed whether defective termination in *ctk1Δ* was dependent on its kinase activity. A kinase independent role for Ctk1p in the release of basal initiation factors from elongating polymerase has previously been described [50]. To this end, two Ctk1p mutants were generated by introducing a T338A change in the T-loop and a D324N change in the active site. The first mutation results in reduced kinase activity because Ctk1p cannot be phosphorylated and activated anymore by Cak1p. The second mutation abolishes all kinase activity of Ctk1p *in vitro* [51]. Northern analysis of *SNR13* transcripts revealed readthrough defects to similar levels as in *ctk1Δ* cells only when the kinase activity of Ctk1p is fully impaired in the Ctk1-D324N mutant (Figure 3A). We note that in agreement with a kinase-independent role for Ctk1p in transcription [50], the Ctk1-D324N only partially rescues the growth defect of *ctk1*Δ (Figure 3B).

These results strongly suggest that the readthrough observed at NNS targets is due to an altered CTD phosphorylation pattern in *ctk1Δ* cells. Although Ctk1p has been shown to affect Ser2 phosphorylation *in vivo* [52], subtle effects on the other phosphoisoforms might occur that impact termination. Indeed it has been proposed that recruitment of Nrd1 depends on the interaction with Ser5 phosphorylated Pol II CTD [21], [23]. However, western blot analysis of whole cell extracts show a major decrease of Ser2P levels, possibly elevated Ser5P levels and unchanged Ser7P levels (Figure S4A, Figure S6).

**Figure 4:**
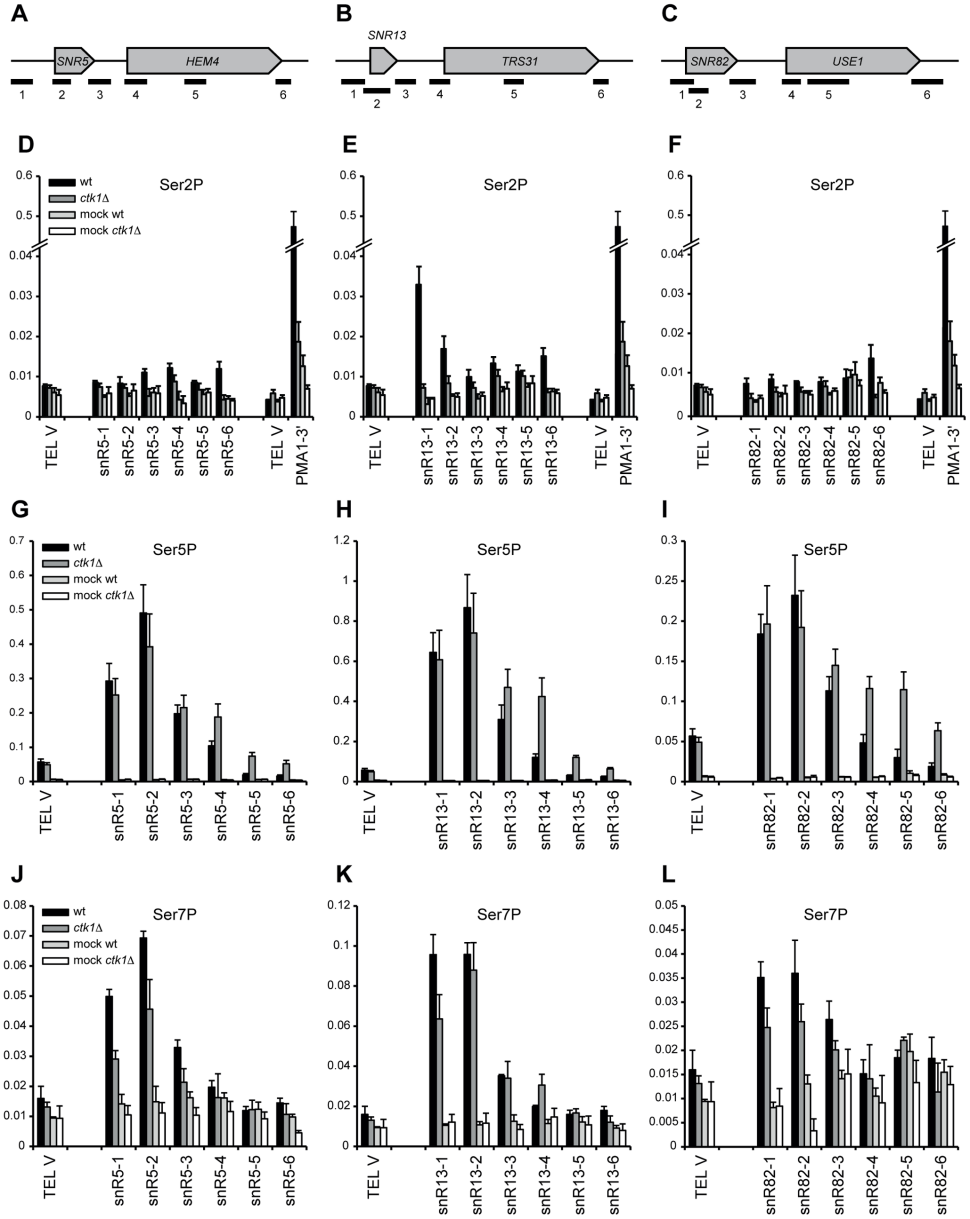
CTD phosphorylation patterns at snoRNAs are not substantially changed in *ctk1*Δ cells. (A, B, and C) Positions of the genomic regions analyzed by ChIP-qPCR. (D, E, and F) ChIP analysis of CTD-Ser2P in wt and *ctk1*Δ at *snR5*, *snR13* and *snR82*. The 3′ end of *PMA1* is used as a positive control. (G, H, and I) ChIP analysis of CTD-Ser5P in wt and *ctk1*Δ at *snR5*, *snR13* and *snR82*. (J, K and I) ChIP analysis of CTD-Ser7P in wt and *ctk1*Δ at *snR5*, *snR13* and *snR82*. All ChIP values (average of 3 biological replicates) represent the fold enrichment, expressed as percent fraction of input. Error bars represent standard error. A telomeric region from chromosome V (*TEL V*) is used as a negative control.

CTD phosphorylation patterns were also examined at individual snoRNAs by ChIP. Ser2P is low at snoRNA sites in wt, as previously observed in genome-wide experiments (Figure 4D–F) [10], and strongly reduced in *ctk1Δ* cells. The 3′ end of *PMA1* is used as a positive control and shows high Ser2P enrichment that is lost upon deletion of *CTK1*. Levels of Ser5P are slightly elevated downstream of the snoRNA genes in *ctk1*Δ (Figure 4G–I), which can be explained by increased Pol II density, possibly due to readthrough. It is therefore unlikely that changes in the distribution of Ser5 phosphorylation explain the readthrough observed in *ctk1Δ*. Levels of Ser7P are slightly decreased (Figure 4J–L), but a role for Ser7P in transcription termination has not been identified.

Upon deletion of Ctk1, the Ser2P levels are strongly reduced, but are not completely absent. To assess whether the low remaining Ser2P levels can be explained by the redundant action of Bur1p, another CTD kinase that has been implicated in Ser2 phosphorylation [13], we constructed strains expressing the *CTK1* and *BUR1* genes under control of the glucose repressible *GAL1* promoter (*P_GAL_CTK1* and *P_GAL_BUR1*) and combined the two in a double *P_GAL_CTK1/P_GAL_BUR1* strain. Depletion of Ctk1p for 3 hours was effective in preventing Ser2P to similar levels as in a *ctk1Δ* strain (Figure S4B). Co-depletion of both Ctk1p and Bur1p only marginally affected the Ser2P signal with respect to Ctk1p depletion alone (Figure S4B), which indicates a minor (if any) role of Bur1p in global CTD-Ser2P.

We also assessed the effect of depletion of Ctk1p on NNS-dependent termination, as well as the effect of double Ctk1p and Bur1p depletion to consider the occurrence of partial redundancy in Ser2P, either under normal conditions or specifically in the absence of Ctk1p. Transcriptional readthrough leading to the production of fusion transcripts terminating at a downstream CPF/CF site were clearly observed upon Ctk1p depletion (Figure S2D). Longer unstable transcripts, likely terminating at downstream sites by the NNS pathways were also observed, the levels of which were not further increased by Bur1p co-depletion, suggesting a minor role for Bur1p in NNS-dependent termination (Figure S2D). These results also verify that readthrough is not an indirect effect of the strong growth defect of *ctk1Δ*.

**Figure 5:**
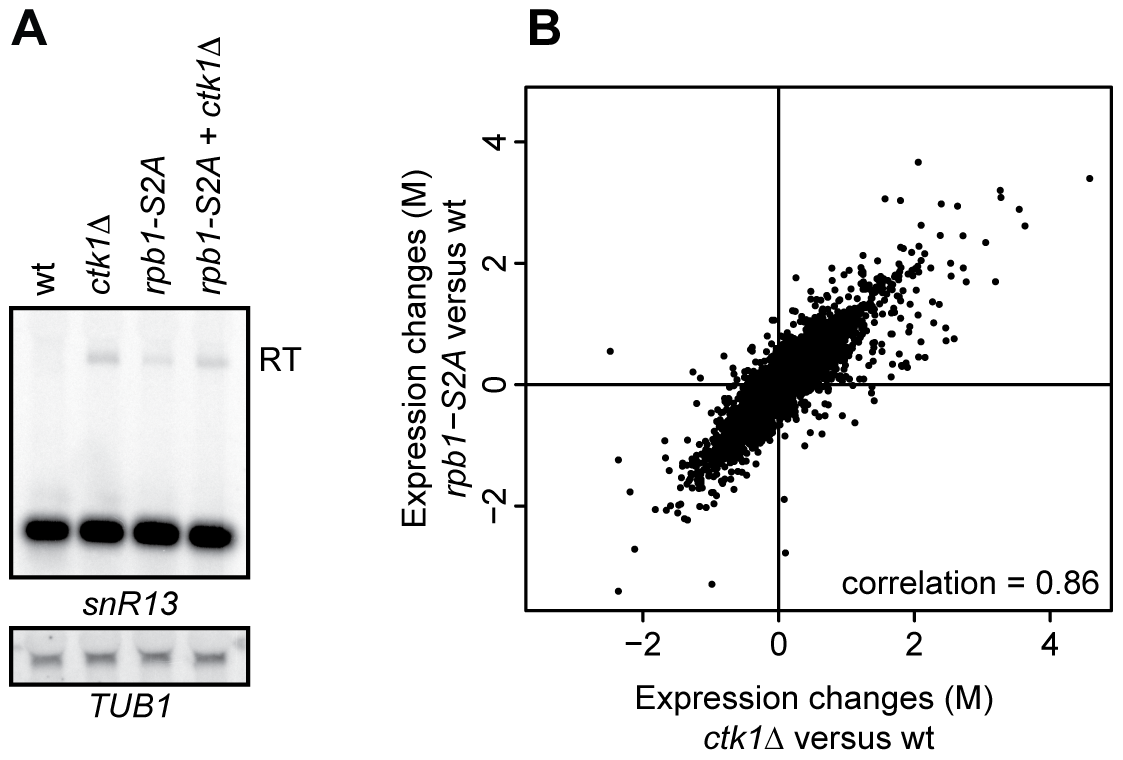
Readthrough is partially explained by Ser2 phosphorylation. (A) Northern blot analysis of an *rpb1*-*S2A* mutant in which every Ser2 in the CTD is mutated to an alanine. Readthrough transcripts (RT) are indicated. (B) Correlation plot of expression changes (M, log2 fold change) in *ctk1*Δ and *rpb1-S2A* versus wt, as measured by microarray analysis.

NNS-dependent termination was only partially impaired in *ctk1Δ* cells. This could occur because residual levels of CTD-Ser2P in *ctk1Δ*, possibly generated by another pathway, are sufficient for basal termination levels. Therefore we analyzed the efficiency of the NNS pathways in a *rbp1-S2A* mutant, in which Ser2 in every repeat was mutated to alanine [53]. *rpb1*-*S2A* cells show clear readthrough at *SNR13*, to similar levels (or even lower) than in *ctk1*Δ cells (Figure 5A). A double *ctk1Δ*/*rpb1*-*S2A* mutant behaved similarly to the single *ctk1*Δ mutant. This indicates that Ser2P impacts the efficiency of NNS-dependent termination but is not absolutely required. Finally, we also considered the possibility that low CTD-Ser2P levels might be the reason why mRNA termination is not globally affected in *ctk1Δ* cells. However, the correlation between the expression profile of *ctk1*Δ and *rpb1*-*S2A* is high (0.86, Figure 5B), showing that the overall expression effects are highly similar and that, similarly to deletion of Ctk1p, the *rpb1-S2A* mutant does not have a global termination defect at mRNAs genes.

The above data strongly suggest that CTD-Ser2 phosphorylation is the primary target of Ctk1p in promoting termination at NNS-dependent genes. They also confirm that CTD-Ser2P impacts the efficiency of NNS-dependent termination but is generally not required for termination of mRNA coding genes.

### Nrd1p recruitment is not affected in *ctk1*Δ

**Figure 6:**
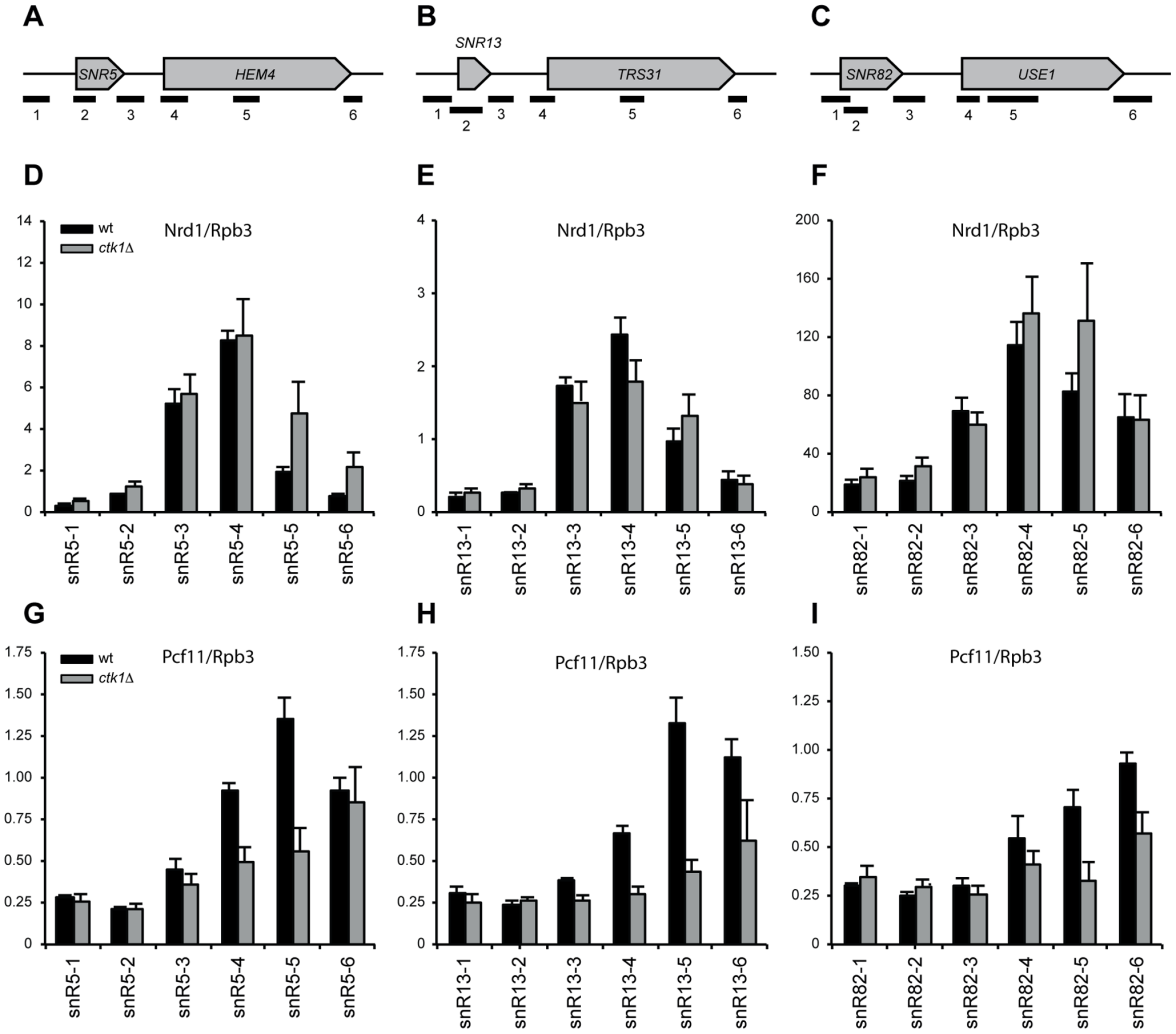
Recruitment of Nrd1p and Pcf11p, relative to Rpb3p. (A, B, and C) Scheme of the genomic regions analyzed by ChIP-qPCR. (D, E, and F) Analysis of Nrd1p occupancy relative to Rpb3p occupancy as determined by ChIP in wild type and *ctk1*Δ strains at the *snR5*, *snR13* and *snR82* loci. Relative ChIP values are normalized to the TEL-V signal in wt. (G, H, and I) Analysis of Pcf11p occupancy relative to Rpb3p occupancy in wt and *ctk1*Δ at *snR5*, *snR13* and *snR82*. Relative ChIP values are normalized to the TEL-V signal in wt. Error bars represent standard errors. Raw Rpb3p, Nrd1p and Pcf11p levels are shown in Figure S5.

Termination defects have previously been associated with aberrant recruitment of termination factors [54]. In order to understand the mechanism by which Ctk1p contributes to the early termination pathway, binding of Pol II, Nrd1p and Pcf11p was determined by chromatin immunoprecipitation (ChIP) at *SNR5*, *SNR13* and *SNR82* (Figure 6 and S5). Pol II levels are increased downstream of all three snoRNAs in *ctk1*Δ, consistent with readthrough into adjacent genes (Fig. S5D–F). As expected, Nrd1p is recruited to the 3′ end of all three snoRNAs in wild type (Fig. S5G–I). Upon deletion of *CTK1*, Nrd1p occupancy closely follows polymerase levels, being unchanged in the body of the genes and elevated downstream (Figure S5G–I; better visible when Nrd1p signals are normalized to Rpb3p signals (Figure 6D–F). Interestingly, we also detected an increased association of Nrd1p with Ser5P Rbp1p in *ctk1Δ* cells by co-immunoprecipitation (Figure S6), which could either be a consequence or a cause of the termination defect (see discussion).

A different pattern is observed for Pcf11p. We observed recruitment of Pcf11p to snoRNA genes that was highest in the termination region. The Pcf11p ChIP signal was seemingly similar in wild type and *ctk1Δ* cells (Figure S5J–L). However, when the signal was considered relative to the level of transcription in each region, i.e. when normalized to Rpb3p signal (that is higher in *ctk1Δ* cells due to the readthrough, Figure S6G–I), Pcf11p occupancy was clearly found to be negatively affected by deletion of Ctk1p, which mirrors Ctk1p dependency at mRNA genes [26]. From these experiments we conclude that defective recruitment of Nrd1p cannot account for the termination defect observed in *ctk1Δ* strain. The results also indicate that Pcf11p (and presumably the CPF/CF complex) is recruited at snoRNA genes in a Ctk1p-dependent manner.

### Partial suppression of the *ctk1Δ* phenotype by a transcription elongation mutant

**Figure 7:**
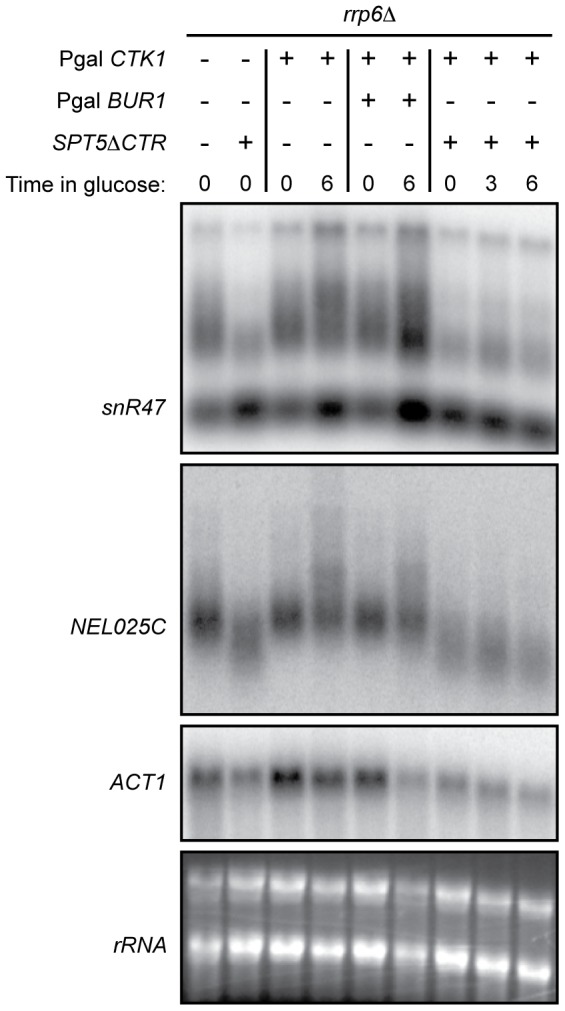
Partial suppression of *ctk1Δ* termination phenotype by a *spt5*Δ*CTR* mutant. Northern blot showing the effect of the *spt5*Δ*CTR* mutation on termination at *snR47* and the *NEL025c* CUT in strains metabolically depleted for Ctk1p and Bur1p. *CTK1* and *BUR1* were expressed under control of the *GAL1* promoter and depletion was carried for the times indicated. Note the appearance of longer RNA species upon depletion of *CTK1* that are suppressed in an *spt5*Δ*CTR* background. An *rrp6Δ* background was used to allow detection of unstable RNA species. Transcripts were revealed with double stranded probes that span the entire *snR47*, *NEL025c* genes or the 3′ end of *ACT1*.

The observation that longer transcripts, presumably representing primary termination products, can be observed at NNS-dependent transcription units in *ctk1Δ* cells, suggests that transcription termination is delayed in the absence of functional Ctk1p. It has recently been shown that termination is sensitive to the elongation properties of RNA pol II, with “faster” polymerase mutants delaying termination and which is suggested to depend on the existence of kinetic competition between transcription and termination [55]. This suggests that in *ctk1Δ* cells, presumably because of the low or absent Ser2P levels, RNA polymerase might behave as a fast mutant with regard to termination. We therefore investigated whether altering the elongation properties of RNA Pol II might impact the delayed termination phenotype of *ctk1Δ* cells. Spt5p is an elongation factor that has an important role in regulating the processivity of RNA Pol II [56]. Its C-terminal region has been implicated to be involved in the recruitment of the PAF complex, another elongation complex. Deletion of the Spt5p C-terminal region (*spt5ΔCTR*) is lethal in a *ctk1Δ* strain. We therefore introduced the *spt5ΔCTR* mutation in a P_Gal_ *CTK1* strain and analyzed the effects of Ctk1p depletion in an *spt5ΔCTR* context. We performed these experiments in an *rrp6Δ* background to visualize CUTs or snoRNA precursors as before. The *spt5ΔCTR* mutation alone produced slightly shorter precursors at most NNS-dependent genes (Figure 7), consistent with the notion of kinetic competition between transcription elongation and termination. Interestingly, the *spt5ΔCTR* mutation suppressed the delayed termination phenotype observed at *NEL025C* and *SNR47* genes upon Ctk1p depletion. Indeed, the upshift of the transcript smear observed upon Ctk1p depletion (compare lanes 4 to 9) is not observed in the *spt5ΔCTR* context.

## Discussion

The results show that removal of the CTD-Ser2P kinase Ctk1 results in readthrough at many snoRNAs, CUTs, SUTs and some mRNAs. Although several mRNAs show readthrough, there is no global defect in mRNA termination. The possibility that this is caused by residual Ser2P has been ruled out, since the *rpb1-S2A* mutant shows similar expression changes as *ctk1*Δ, lacking extensive additional changes compared to *ctk1*Δ that would be expected upon a global termination defect. These results indicate that Ser2P is not essential for the late termination pathway, but has in fact a more prominent role in the early termination pathway than previously anticipated. The current model that the early termination pathway is dependent on CTD-Ser5P and the late termination pathway on Ser2P [21], [23] therefore appears to be too simplistic.

The finding that Ser2P is not essential for mRNA termination by the CPF/CF complex is surprising, since previous studies have demonstrated that several of its subunits (e.g. Rna14p, Rna15p and Pcf11p) are recruited in a Ctk1p- (and Ser2P-) dependent manner. Binding of Pcf11p and another termination factor, Rtt103p, to the CTD has clearly been shown to be dependent on Ser2P on the CTD [16], [20]. The fact that mutation of these factors has massive effects on termination [48], [49] is seemingly at odd with the poor effect of Ser2P absence, but might suggest that the CTD has a redundant (and generally minor) role in the recruitment of the CPF/CF and possibly in the termination process. This role might be revealed by “sensitive” RNAs as already suggested by Kim and colleagues [11], which is consistent with our results. Recruitment of the CPF/CF might be more strongly dependent on the nascent RNA than on CTD-Ser2P binding. We suggest that the CPF/CF complex is more efficiently crosslinked to the DNA when bound to the CTD than when associated to the nascent RNA, which explains why the apparent loss of recruitment is not accompanied by a major termination defect.

The extensive readthrough observed at NNS-terminated genes is indicative of an important role of Ctk1p in termination at these targets. We observed a somewhat stronger effect in *ctk1Δ* or in kinase dead cells than in S2A cells, in which every Ser2 in the CTD has been mutated to alanine. Although this might suggest that Ctk1p also exerts its role in NNS-dependent termination via the phosphorylation of other substrates, the clear effect of the S2A mutation, both on model substrates and genome-wide, indicates a positive role of the CTD-Ser2P phosphoisoform. Previously, several genome-wide studies have shown that Ser2P is low in the early phases of transcription, i.e. at positions where NNS termination occurs [10], [11], [57]. Similarly, Ser2P is also very low at snoRNAs (Fig. 4 and [10]). Moreover, the NNS complex, via its subunit Nrd1p recognizes the Ser5P form of the CTD most efficiently [21]. This is an apparent conundrum that needs to be resolved.

It is theoretically possible that Ctk1p affects a minor component of termination at snoRNAs and CUTs that is CPF-dependent. Consistently, we and others have recently shown that the NNS- and the CPF/CF pathway can recognize very similar signals and the choice of the pathway is strongly dependent on the position of the signals relative to the transcription start site [23], [24], [58], [59]. Thus low levels of recognition by the “wrong” pathway might occur at most terminators, but be masked by the prevalence of the other pathway (NNS in the case of snoRNAs and CUTs).

Two observations, however, argue against this model. The first is that transcripts terminated by the CPF/CF pathway are expected to be relatively stable and should be observed at the primary termination sites for at least some of the snoRNAs or CUTs in a wild type strain, which is generally not the case (Fig. 1C–E, S1). The other conflicting observation is that only these putative CPF-dependent transcripts should disappear in a *ctk1Δ* strain to give longer transcripts. Instead, we observe a clear upshift in size of the whole set of snoRNAs precursors (or CUTs) analyzed in this context, most of which are unstable and NNS-dependent (Fig. 1C–E, S1 and S2C).

Our observations favor a role for Ctk1p and CTD-Ser2 phosphorylation in the NNS pathway. Considering the number of transcripts affected and the specificity of the effect, it is unlikely that Ctk1p mutation affects NNS termination indirectly. Although it is clear that the impact of Ctk1p in this pathway remains minor compared to the role of the NNS complex, it is functionally significant.

How does phosphorylation of the CTD at Ser2 impact NNS termination? One possibility is that the onset of Ser2P, occurring relatively early in transcription [57], is required for the efficiency of the process. For instance it is possible that a low level of Ser2P is required for the release of Nrd1p from the CTD, which, in turn, triggers termination. We show that the Nrd1p ChIP signal downstream of snoRNA terminators is higher in *ctk1Δ* cells than in the wild type and that the association of Nrd1p with the polymerase is increased in the mutant (Fig. S5G–I, S6). These findings could be a consequence of defective termination, i.e. due to the persistent association of Nrd1p and polymerase during readthrough transcription. However, it is also possible that the dissociation of Nrd1p from the polymerase is required for an efficient transcription termination reaction, as previously suggested to explain the mechanism of Ess1p action in the process [30]. It can be envisioned that a low level of Ser2P would favor this step by inherently weakening the interaction of the CTD with the Nrd1 CID or by recruiting Pcf11p, which would compete with Nrd1p binding thereby facilitating its release. This would be consistent with the observed decrease of Pcf11p occupancy at snoRNAs in *ctk1Δ* cells (Figures 6 and S5), although we could not suppress the *ctk1Δ* termination defect by Pcf11p overexpression (data not shown).

Alternatively, Ser2P could affect the processivity or the speed of the polymerase and progressively induce a termination mode that favors the function of the CPF/CF complex at the end of mRNA genes. This effect might be already detected in a termination-sensitized context at NNS terminators. This could easily explain the delayed termination phenotype (of which the readthrough might be the extreme consequence) in *ctk1Δ* cells and its suppression by the *spt5ΔCTR* mutant (Fig. 7). Delayed termination at NNS sites has been recently described using “fast” mutational variants of RNA polymerase (*rpb1-E1103G*) and interpreted to indicate kinetic competition between termination and elongation [55]. Indeed, the *rpb1-E1103G* mutation was shown to aggravate the termination phenotype of *sen1* mutants [55]. We suggest that in *ctk1Δ* cells (or a S2A-CTD Rpb1p mutant) the absence of Ser2P translates into Pol II transcriptional properties that resemble those of a “fast” Pol II mutant. The *spt5ΔCTR* mutant might suppress the delayed termination phenotype by impacting the transcription properties of the Ser2P-less polymerase. The results presented in this study indicate that further mechanistic analyses of Ctk1 and CTD-Ser2P are both required and will likely be rewarding, given the altered role that the results demonstrate for both.

## Supporting Information

Figure S1 **Loss of** ***CTK1*** **results in readthrough at snoRNAs.** (A, B and C) Northern blot analysis of *snR43*, *snR82*, and *snR60* transcripts in *ctk1*Δ and *sen1-1* cells The positions of the probes are indicated above the Northern blots. Readthrough transcripts (RT) and precursor transcripts are indicated. RT species are detected with probes in the downstream genes. *TUB1* mRNA was used as a loading control.
(TIF)

Figure S2 **Loss of** ***CTK1*** **results in readthrough at CUTs, SUTs and small ORFs.** Northern blot analysis of transcripts derived from (A) *SER3*, (B) *BIO5* and (C) *CUT882*. Readthrough transcripts (RT) and longer unstable transcripts are indicated. Note that RT transcripts at the *CUT882* locus are not efficiently detected by northern blot unless degradation is also impaired in the *ctk1*Δ/*rrp6*Δ double mutant. (D) Northern blot analysis of *NEL025c*, *NGR060w* CUTs and *snR33* precursors in strains metabolically depleted for Ctk1p and/or Bur1p in Pgal-*CTK1* and Pgal-*BUR1* strains. An *rrp6Δ* background was used to allow detection of unstable RNA species. Transcripts were revealed with double stranded probes that span the entire gene. (E–F) Metagene analysis displaying the average probe intensity over 25 bp regions in wt and *ctk1*Δ, aligned by the 3′ end of small (<600 bp) or long (>3000 bp) ORFs.
(TIF)

Figure S3 ***ctk1*** **Δ cells are not generally defective for mRNA termination.** Northern blot analysis of *CYC1*, *ACT1* and *ARO8* RNAs in a *ctk1*Δ strain. Thermosensitive mutants of the CPF/CF complex (*rna14-3* and *rna15-2*) were used for comparison. The analysis was also performed in an *rrp6Δ* background to allow detection of unstable RNA species (right). Note the presence of high levels of readthrough transcripts that are partially unstable in CPF/CF mutants at the non-permissive temperature (2 hours at 37°C). Normally terminated RNAs are instead produced in *ctk1Δ* cells.
(TIF)

Figure S4 **Loss of Ctk1p mainly affects the levels of Rpb1 CTD-Ser2P.** (A and B). Western blot analysis showing the levels of the different Rpb1 CTD phosphoisoforms in a *ctk1Δ* cells (A) and in conditions of metabolic depletion of Ctk1p and/or Bur1p (B). Depletions of Ctk1p and Bur1p was obtained by growth of *Pgal-CTK1* or *Pgal-BUR1* strains in the presence of glucose for 6 hours.
(TIF)

Figure S5 **Recruitment of termination factors at snoRNA termination sites in** ***ctk1*** **Δ.** (A, B, and C) Positions of the genomic regions analyzed by ChIP-qPCR. (D, E, and F) ChIP analysis of Rpb3 in wt and *ctk1*Δ at *snR5*, *snR13* and *snR82*. ChIP values represent the average of 3 biological replicates as in fig. 4. (G, H, and I) Nrd1p occupancy at *snR5*, *snR13* and *snR82* in wt and *ctk1*Δ cells. Average of 4 biological replicates. (J, K and I) Pcf11-TAP occupancy at *snR5*, *snR13* and *snR82* in wt and *ctk1*Δ. Average of 2 biological replicates. All ChIP values represent percent enrichment *TEL V* is used as a negative control. Nrd1p and Pcf11p levels relative to Rpb3p are shown in Figure 6.
(TIF)

Figure S6 **Nrd1p association with Rpb1p is higher in** ***ctk1*** **Δ cells.** Co-immunoprecipitation experiment using Nrd1p-TAP as bait. Associated proteins are revealed by western blot using specific antibodies as indicated. Immunoprecipitation was performed in the presence or absence of RNase A. An increased association of Nrd1p with RNA Pol II and specifically the CTD-Ser5P form was consistently observed. Dashed line indicates removal of lanes from blot.
(TIF)

Table S1 **List of genes in the clusters of** **Figure 1A** **.**
(XLSX)

Table S2 **Yeast strains.**
(XLS)

Table S3 **Northern probes and ChIP primers.**
(XLS)
