# Supplementary figures and images for: The Role of Ctk1 Kinase in Termination of Small Non-Coding RNAs
Source: PLoS One. 2013 Dec 4;8(12):e80495. doi: 10.1371/journal.pone.0080495 (PMC3851182; doi:10.1371/journal.pone.0080495)

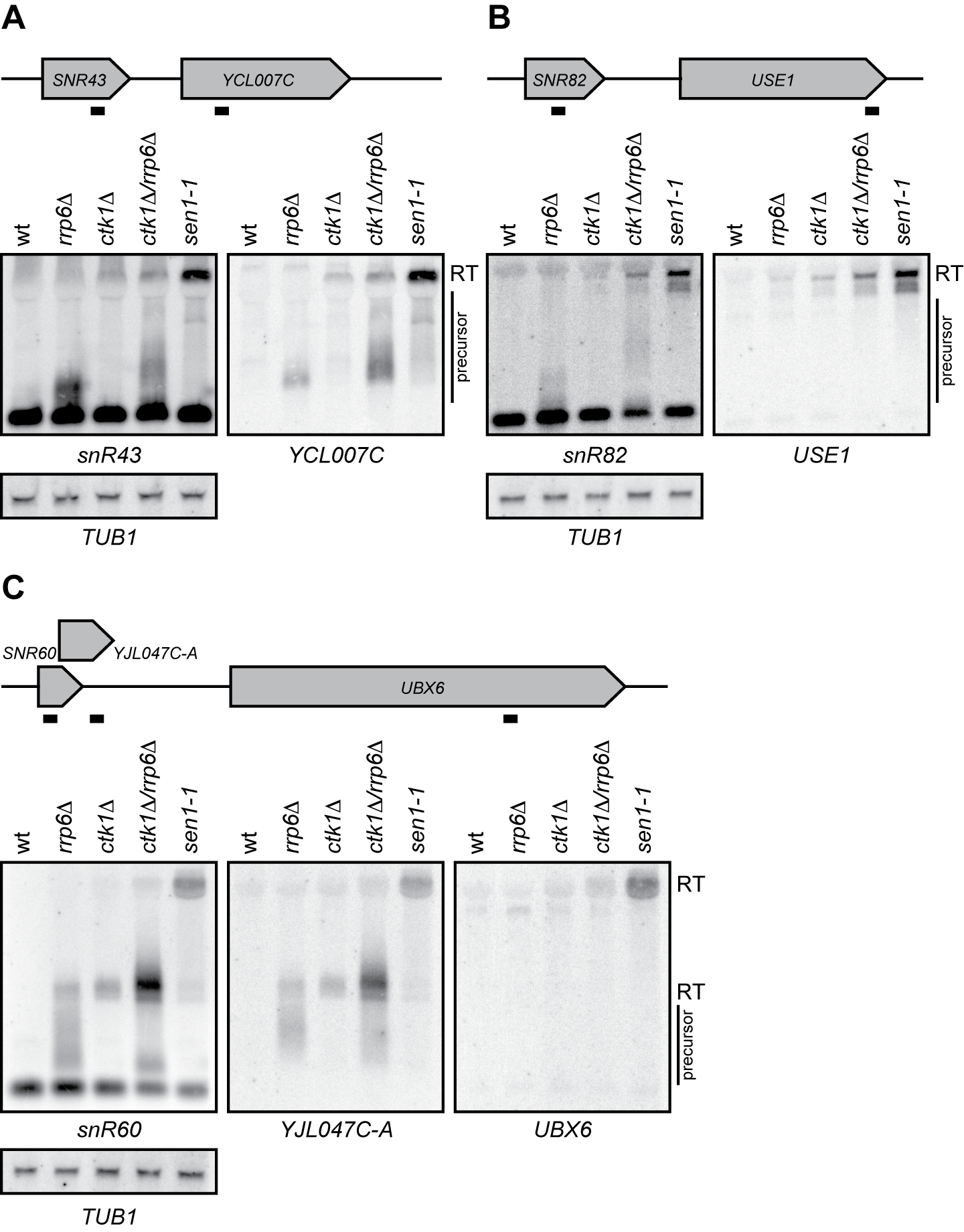

Supplement: Figure S1 — Loss of CTK1 results in readthrough at snoRNAs. (A, B and C) Northern blot analysis of snR43, snR82, and snR60 transcripts in ctk1Δ and sen1-1 cells The positions of the probes are indicated above the Northern blots. Readthrough transcripts (RT) and precursor transcripts are indicated. RT species are detected with probes in the downstream genes. TUB1 mRNA was used as a loading control. (TIF) [file pone.0080495.s001.tif]

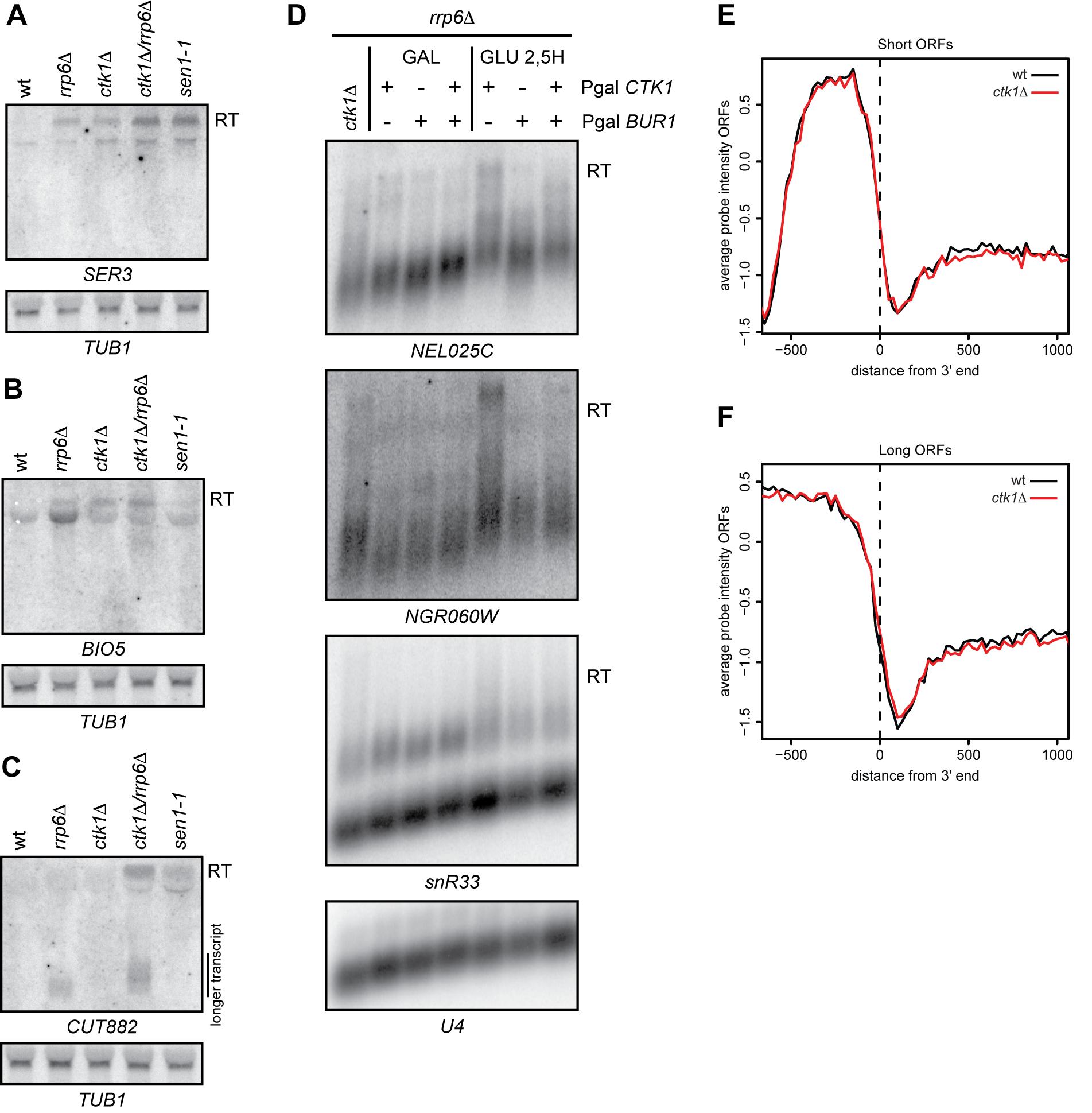

Supplement: Figure S2 — Loss of CTK1 results in readthrough at CUTs, SUTs and small ORFs. Northern blot analysis of transcripts derived from (A) SER3, (B) BIO5 and (C) CUT882. Readthrough transcripts (RT) and longer unstable transcripts are indicated. Note that RT transcripts at the CUT882 locus are not efficiently detected by northern blot unless degradation is also impaired in the ctk1Δ/rrp6Δ double mutant. (D) Northern blot analysis of NEL025c, NGR060w CUTs and snR33 precursors in strains metabolically depleted for Ctk1p and/or Bur1p in Pgal-CTK1 and Pgal-BUR1 strains. An rrp6Δ background was used to allow detection of unstable RNA species. Transcripts were revealed with double stranded probes that span the entire gene. (E–F) Metagene analysis displaying the average probe intensity over 25 bp regions in wt and ctk1Δ, aligned by the 3′ end of small (<600 bp) or long (>3000 bp) ORFs. (TIF) [file pone.0080495.s002.tif]

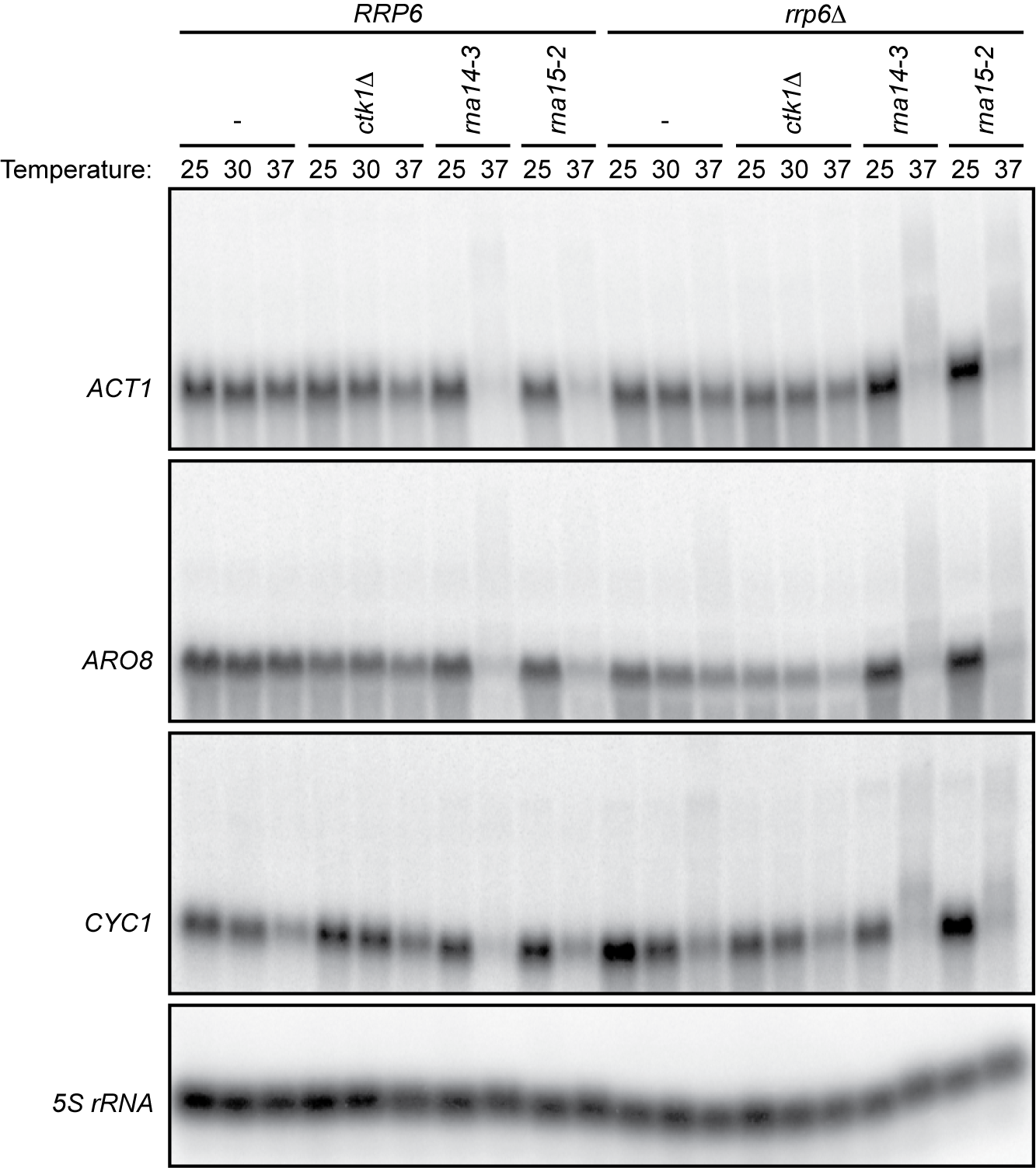

Supplement: Figure S3 — ctk1 Δ cells are not generally defective for mRNA termination. Northern blot analysis of CYC1, ACT1 and ARO8 RNAs in a ctk1Δ strain. Thermosensitive mutants of the CPF/CF complex (rna14-3 and rna15-2) were used for comparison. The analysis was also performed in an rrp6Δ background to allow detection of unstable RNA species (right). Note the presence of high levels of readthrough transcripts that are partially unstable in CPF/CF mutants at the non-permissive temperature (2 hours at 37°C). Normally terminated RNAs are instead produced in ctk1Δ cells. (TIF) [file pone.0080495.s003.tif]

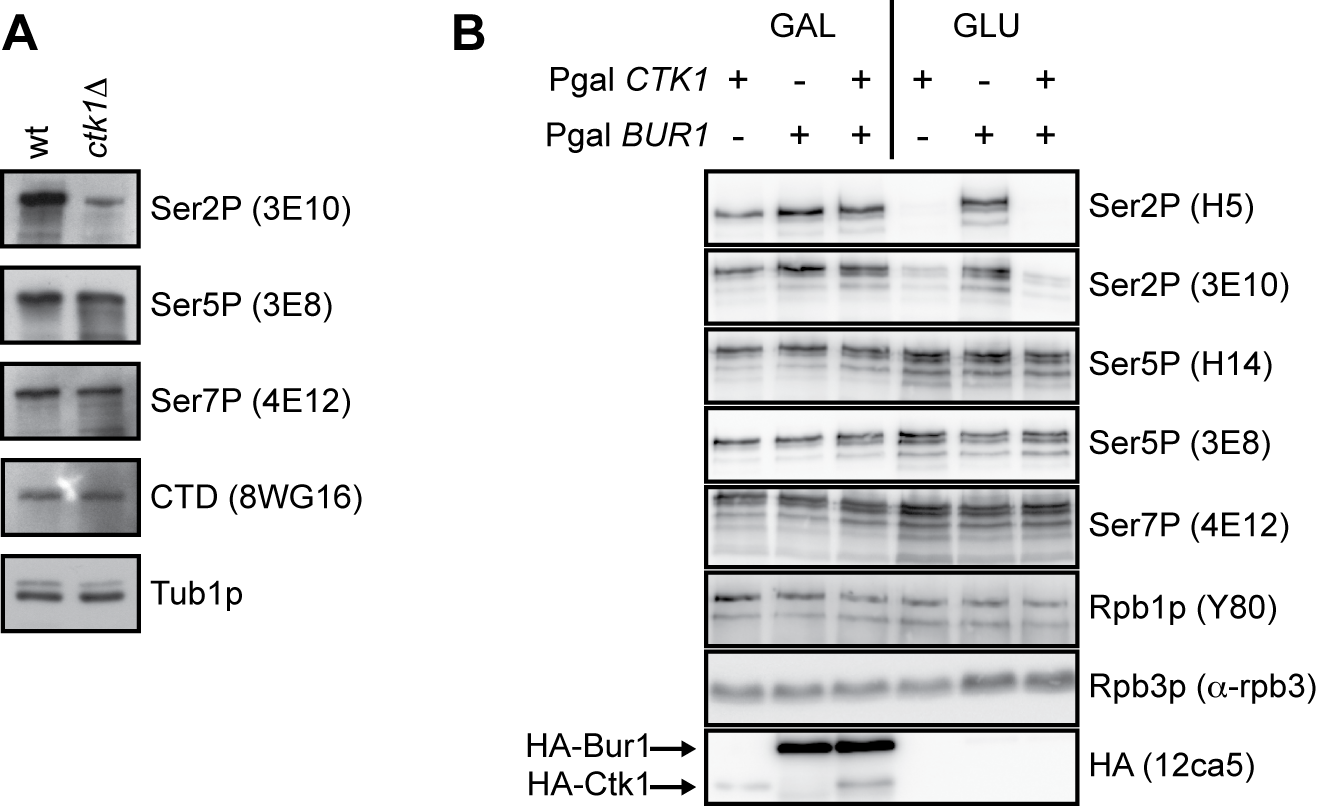

Supplement: Figure S4 — Loss of Ctk1p mainly affects the levels of Rpb1 CTD-Ser2P. (A and B). Western blot analysis showing the levels of the different Rpb1 CTD phosphoisoforms in a ctk1Δ cells (A) and in conditions of metabolic depletion of Ctk1p and/or Bur1p (B). Depletions of Ctk1p and Bur1p was obtained by growth of Pgal-CTK1 or Pgal-BUR1 strains in the presence of glucose for 6 hours. (TIF) [file pone.0080495.s004.tif]

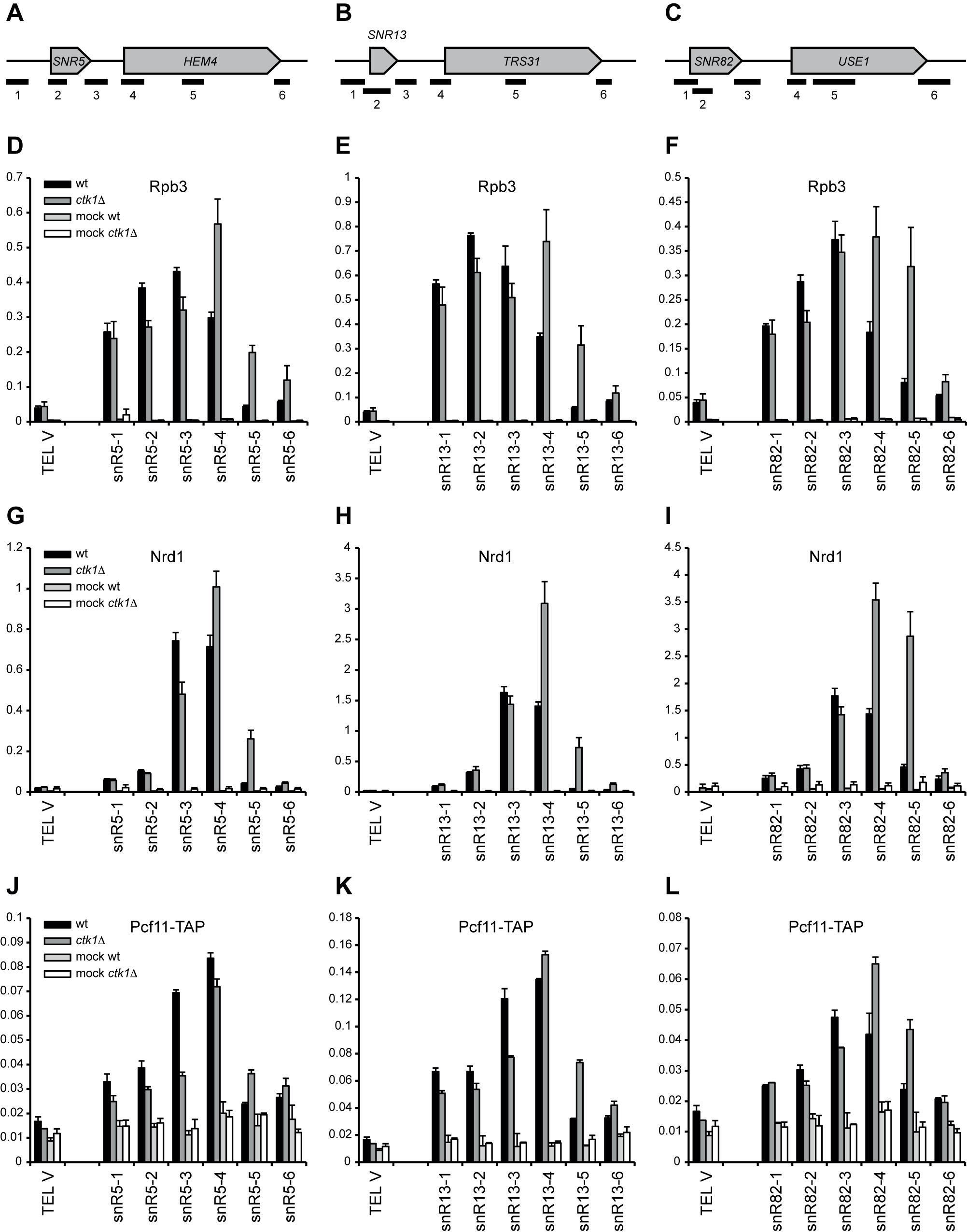

Supplement: Figure S5 — Recruitment of termination factors at snoRNA termination sites in ctk1 Δ. (A, B, and C) Positions of the genomic regions analyzed by ChIP-qPCR. (D, E, and F) ChIP analysis of Rpb3 in wt and ctk1Δ at snR5, snR13 and snR82. ChIP values represent the average of 3 biological replicates as in fig. 4. (G, H, and I) Nrd1p occupancy at snR5, snR13 and snR82 in wt and ctk1Δ cells. Average of 4 biological replicates. (J, K and I) Pcf11-TAP occupancy at snR5, snR13 and snR82 in wt and ctk1Δ. Average of 2 biological replicates. All ChIP values represent percent enrichment TEL V is used as a negative control. Nrd1p and Pcf11p levels relative to Rpb3p are shown in Figure 6. (TIF) [file pone.0080495.s005.tif]

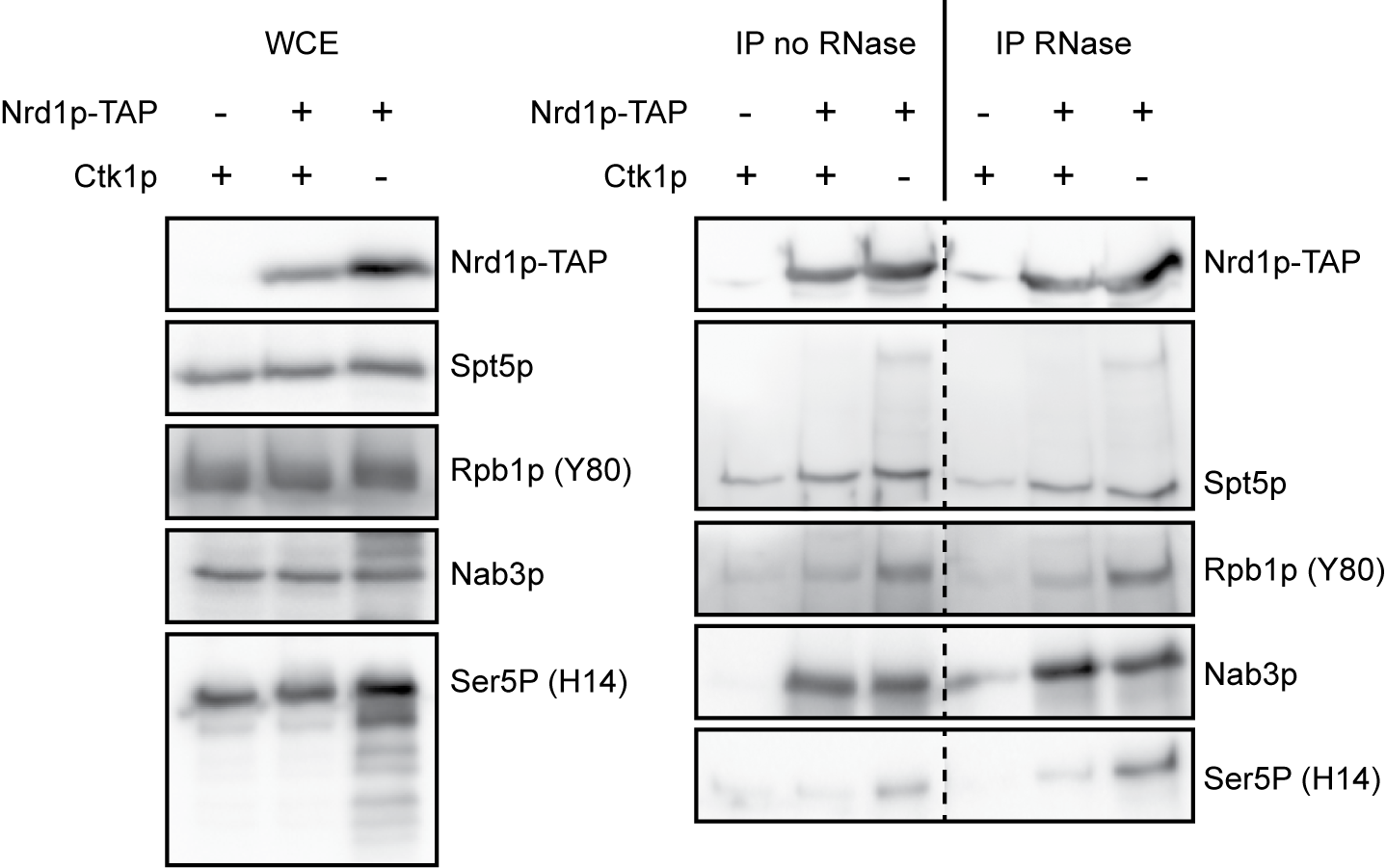

Supplement: Figure S6 — Nrd1p association with Rpb1p is higher in ctk1 Δ cells. Co-immunoprecipitation experiment using Nrd1p-TAP as bait. Associated proteins are revealed by western blot using specific antibodies as indicated. Immunoprecipitation was performed in the presence or absence of RNase A. An increased association of Nrd1p with RNA Pol II and specifically the CTD-Ser5P form was consistently observed. Dashed line indicates removal of lanes from blot. (TIF) [file pone.0080495.s006.tif]
